# Viral Aetiology in Adults with Acute Upper Respiratory Tract Infection in Jinan, Northern China

**DOI:** 10.1155/2013/869521

**Published:** 2013-04-15

**Authors:** Yanqin Lu, Jiabei Tong, Fengyan Pei, Yanping Yang, Dong Xu, Mingyu Ji, Chunyan Xing, Pingdong Jia, Chao Xu, Yunshan Wang, Gongchao Li, Zhenbin Chai, Yan Liu, Jinxiang Han

**Affiliations:** ^1^Shandong Medicinal Biotechnology Centre, Key Laboratory for Modern Medicine and Technology of Shandong Province, Key Laboratory for Virology of Shandong Province, Key Laboratory for Biotech-Drugs, Ministry of Health, Shandong Academy of Medical Sciences, Jinan 250062, China; ^2^Central Laboratory, Affiliated Jinan Central Hospital of Shandong University, Jinan 250013, China; ^3^Department of Respiratory, Affiliated Jinan Central Hospital of Shandong University, Jinan 250013, China

## Abstract

Our study investigated the epidemiology of respiratory viruses in adult patients with upper respiratory tract infection (URTI) between August 2009 and September 2010 in Jinan, northern China. Nasal and throat swabs (*n* = 596) were collected from adult patients with URTIs. Nine respiratory-related viruses, including IFV, PIV, HRV, HMPV, HBoV, HCoV, ADV, RSV, and EV, were detected in all samples by conventional and reverse transcription polymerase chain reactions. Positive detection rate for respiratory virus was 38.76% and codetection rate was 4.70% in adults with acute respiratory tract infections. IFV (20.81%) was the dominant agent detected and IFVB had a higher incidence (12.58%) than IFVA (7.72%). Detection rates of 8.22%, 5.03%, 3.69%, and 2.52% were observed for HBoV, HRV, EV, and RSV, respectively. HCoV had the lowest detection rate of 0.50%. HBoV, HRV, EV, and ADV infection rates were higher in the 14–25-year-old group than in the 26–65-year-old group. Codetection rates were higher (7.52%) in the 14–25-year-old group than in the older age group (2.64%). The spectrum of respiratory virus infection in adult patients with URTIs was different in Jinan compared with other cities in China.

## 1. Introduction

Acute respiratory tract infections (ARTIs) are a severe public health issue throughout the world and the leading cause of paediatric morbidity [1]. Viruses that are closely associated with ARTIs include respiratory syncytial virus (RSV), parainfluenza viruses (PIVs), influenza viruses (IFVs), enteroviruses (EVs), adenoviruses (ADVs), human rhinoviruses (HRVs), and human coronaviruses (HCoVs) 229E and OC43. Associated novel viruses have also been reported, such as severe acute respiratory syndrome coronavirus [2], human bocavirus (HBoV) [3], and WU and KI polyomaviruses [4].

Most studies of ARTI-related viruses have focused on children [5, 6]. RSV is reported as the major pathogens for lower respiratory tract infections in hospitalised children, while various other ARTI-associated pathogens have also been reported, including RV, HMPV, HBoV, and PIV [7, 8]. Only limited data have been reported for adult patients. Assessment of viral infection prevalence in adults in China based on a large population study has only been reported for Beijing and Shanghai [9–12]. We, therefore, aimed to investigate the viral aetiology in adult patients with ARTIs in Jinan, a city in northern China, between August 2009 and September 2010.

## 2. Materials and Methods

### 2.1. Clinical Specimens

During the period between August 2009 and September 2010, a total of 596 nasal and throat swabs (NTSs) were collected from adult patients with ARTIs at the Jinan Central Hospital affiliated with Shandong University. Separate NTSs were placed into viral transport media. Patients over 14 years enrolled in the study were selected by physicians according to the presence of one or more respiratory symptoms, including watery eyes, rhinorrhea, nasal congestion or sinus congestion, otitis media, pharyngitis, cough, sore throat, sneezing, headache, and muscle pain. Meanwhile, enrolled patients had at least one symptom during acute infection, with high fever (body temperature ≥ 38°C) or chillness or normal/low leukocyte count. A standardised form including symptoms, illness history, and clinical examination was recorded for each patient. Clinical symptoms and blood test of analyzed subjects are summarized in Table 1. Informed consent was obtained from each patient. The study was approved by the Ethics Committee of Shandong Medicinal Biotechnology Centre.

### 2.2. Nucleic Acid Extraction

Total nucleic acids including DNA and RNA were extracted from 200 *μ*L of each specimen using a QIAamp MinElute Virus Spin Kit (Qiagen, Mississauga, ON, Canada) according to the manufacturer's instructions.

### 2.3. PCR/RT-PCR Screening for Respiratory Viruses

ADV, HBoV, HCoV, HMPV, IFV, RSV, PIV, EV, and HRV viruses were detected by conventional polymerase chain reaction (PCR) or reverse transcription- (RT-) PCR assay. PCR was conducted according to previously published studies. Detection of PIV, EV, HRV [13], IFV A, B, and C, and RSV [14] was performed using two multiplex nested RT-PCRs. HCoVs [15] and HMPV [16] were detected using two-step RT-PCRs, and ADV [17] was detected by one-step PCR. HBoV was detected by touch-down PCR as previously reported [18].

RT-PCR was performed using a SuperScript II one-step RT-PCR Platinum Taq kit (Invitrogen, Carlsbad, CA, USA) to obtain cDNA. Conventional PCR was performed using Ex Taq polymerase (Takara, Otsu, Japan). All products were subjected to electrophoresis on a 2% agarose gel. PCR/RT-PCR protocols for each virus were available in detail (see Supplementary 1 available online at http://dx.doi.org/10.1155/2013/869521). 

### 2.4. Statistical Analysis

Chi-square test with a significance level of *P* < 0.05 was conducted to compare infection rates among different patient groups.

## 3. Results

### 3.1. Prevalence of Respiratory Viruses

**Figure 1:**
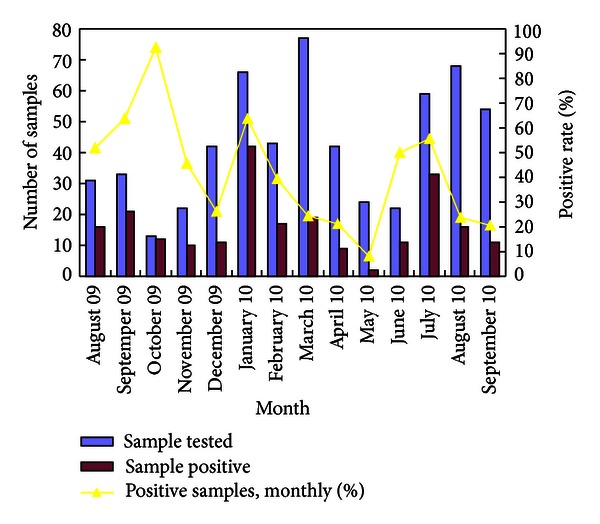
Number of tested and positive patients with ARTIs and the detection rate for each month.

Enrolled patients were aged between 14 and 86 years old (mean age = 31 years). Of 596 patients with URTIs, 231 patients (38.76%) were found to be positive for one or more viruses. The monthly detection rate of enrolled samples ranged from 20.37% to 92.31% (Figure 1). Detection rate of viruses in males and females was 40.55% (118/291) and 37.05% (113/305), respectively, with no significant difference observed between them (*X* = 2; *P* > 0.05). IFV had the highest detection rate (20.81%), followed by HBoV (8.22%), HRV (5.03%), EV (3.69%), and RSV (2.52%). PIV, HMPV, and ADV had the equal low prevalence rates of 0.84%. HCoV was detected at the lowest positive rate (0.67%). 

### 3.2. Seasonality of Respiratory Virus Infection

**Figure 2:**
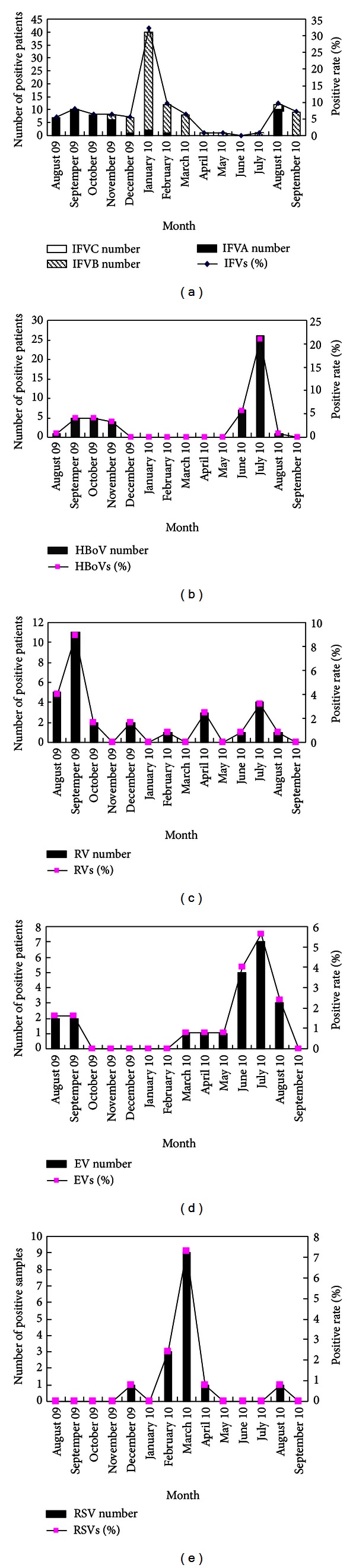
Number of positive patients infected with respiratory virus and the detection rate for each month. (a) IFV. (b) HBoV. (c) RV. (d) EV. (e) RSV.

Seasonal distribution of the different viruses tested was variable and remarkable. IFVB prevalence peaked at the end of autumn, with a detection rate of 43.75% in November 2009, and increased dramatically to 90.48% in January 2010. Infection continued to decrease until the detection rate was 11.11% in April 2010. IFVB was inactive from April to August 2010, after which a second small peak of infection was observed in September 2010, with a detection rate of 81.82%. IFVA was active at the end of summer and throughout spring when it remained at a relative stable prevalence (34.48–50%) from August to November 2009. IFVC was rarely detected, with only three cases observed sporadically during May and October 2010 (Figure 2(a)). 

HBoV was the second most prevalent viral agent, which had obvious seasonally distributed infection rates in summer and autumn. A 20.98% positive rate for HBoV was observed in July 2010. In comparison, a relatively low detection rate (3.23–4.03%) was observed for this virus throughout autumn (Figure 2(b)). 

HRV infections, as the third frequently prevalent virus after HBoV, had no regular seasonality. This virus was found during all months tested except November 2009 and January, March, and May 2010. An epidemic peak was observed in September 2009 (Figure 2(c)).

The seasonal distribution of EV was regular, remaining at a stable detection rate (0.81%) throughout spring before reaching a peak in summer. No cases were observed from October 2009 February 2010 (Figure 2(d)). RSV was detected in December 2009 and in February, March, April and August 2010. This virus had a 7.26% detection rate in March 2010 (Figure 2(e)).

### 3.3. Respiratory Virus and Codetection in Different Age Groups

In the 14–25-year age group, detection rates for HBoV, HRV, EV, and ADV were higher than in the older age groups, especially for HBoV (Table 2).

Codetection with two or more than two different viruses was detected in 28 cases (4.70%). One case was found to have a triple infection of PIV2, IFVB, and HBoV. Twelve different types of mixed viruses were observed (Table 3). Codetection within each of the age groups was different and was significantly more frequent in the 14–25-year-old group (*n* = 20) than in the 26–65-year-old group (8). The most common viruses in these dual infections were HBoV and IFVA, which were codetected in 18 and 10 cases, respectively. IFVA/EV and HBoV codetection were only detected in the 14–25-year-old group, while HRV and HBoV were only detected in the 26–65-year-old age group (Table 3).

## 4. Discussion

Epidemiology and aetiology of respiratory viruses have been extensively studied throughout the world, especially within child populations. Yet study of ARTIs in adult patients is more limited. The prevalence, clinical profile, and epidemiology of respiratory virus in adults are different from those in young people. Also, the relationship between geographical distribution, season, prevalence year, and respiratory viruses is not fully understood in adults. Consequently, there is a clear need to further study the prevalence of respiratory viruses in adults with ARTIs. 

Detection rates of respiratory viruses in adult patients with ARTIs have been variable from report to report. A previous study that investigated IFVA, IFVB, RSV, and HMPV found positive rates of 12.8% in adult patients with ARTIs during the 2001-2002 seasons [20]. We compared data from adults with ARTIs from Beijing, and Shanghai with our own results (Table 4). In Beijing, rates of 52.88% and 34.6% were reported from two different laboratories [11, 19], and a 27.47% positive rate was observed in Shanghai [10]. In comparison, our study observed a 38.76% positive rate in Jinan. The reason for these differences is potentially because of variable PCR detection sensitivity for each of the studies. Detection rates for ADV, HCoV, and HRV/EV by real-time PCR were more than 10 times those detected using conventional PCR. However, there was no major difference between real-time PCR and conventional PCR for the other viruses tested. 

IFV was the leading pathogen detected in adults with ARTIs (positive rate of 20.81%). These data were consistent with the positive rate observed in Beijing. However, IFVB was more prevalent than IFVA in Jinan (12.75% versus 7.55%). Higher incidence of IFVA than IFVB was observed in both Beijing and Shanghai [10, 11, 19]. Our study also highlighted a clear seasonal distribution of IFV. IFVB was active in cold winter and IFVA was active in autumn, while both were rarely detected in summer. 

HBoV has been reported to be associated with respiratory tract disease since 2005, especially in cases associated with children [3, 21]. However, infection in adults was only recorded in a few papers [22–24]. In one report, infection incidence in Guangzhou was 3.39% in children and 0.39% in adults. HBoV infection was also detected at relatively low levels (0.37%) in a group aged 15–49 years, but not in other groups [10]. In comparison, a higher infection rate (8.22%) of this virus was observed in our study. HBoV tended to be more prevalent in the younger age group and had an obvious seasonal preference for summer and autumn. However, further study into its association with clinical characteristics is needed.

HRV was detected as the third main pathogen associated with ARTIs in adults in our study, which was at slightly lower levels than those observed in Beijing (6.5%) [11]. Also, positive rates of HRV in our study were much lower than those reported in children [21, 25]. This may be explained by children being more susceptible to infection than adults. Similarly, RSV was also detected with a low incidence in our study, which was consistent with studies in Beijing, where 0–0.75% positive rates were reported [10, 11, 19]. 

EV incidence in our study was in accordance with that reported in Beijing [11]. A higher prevalence was observed during summer, relatively low levels were found in spring and infection disappeared during the colder months. This virus carries a respiratory tract tropism and is reported predominately in young children. 

In this study, we reported the aetiology of URTIs in adult patients in Jinan city. Limited data have been compared for URTIs with LRTIs in adult patients. IFV is the predominant virus in both URTIs and LRTIs as reported. PIVs were detected at the rate of 7.3% in LRTIs than those of 4.2% in URTIs in Beijing [11]. Picornavirus was reported as the second main pathogen associated with both URTIs and LRTIs in Shanghai, though this data was calculated from both children and adults [10].

We codetected HBoV with IFVA, HRV, EV, and IFVB in the study. The codetection rate of 2.5% in LRTIs and 1.6% in URTIs was reported in adults. The prevalence of mixed respiratory viruses in adults was lower than that in children. The incidence of codetection was 5.29% in children with URTIs and 21.05% with LRTIs in Jinan city (our unpublished data). This supports previous studies that reported a higher incidence of RSV-HMPV and RSV-HRV codetections in children [27, 28]. The clinical significance of mixed respiratory virus remains unclear. One controversial opinion of codetection in children is that MPV-RSV codetections present with more severe symptoms than a single infection [29, 30], although this has been disputed in other reports. While our study did not assess clinical severity, our data suggested that codetection tended to occur more frequently in younger patients than in adults. 

## 5. Conclusions 

In summary, we investigated the prevalence of nine respiratory viruses in adults with upper respiratory tract infections URTIs in Jinan. The spectrum, seasonal distribution, age, and codetections were studied. Some of our findings were similar to those reported by Ren et al. for Beijing and Shanghai, including detection rate of IFV, ADV, and EV [12]. In comparison, the highest positive rates reported by Yu et al. were for ADV, HCoV, and picornavirus [19], which had low positive rates in our study (Table 4). Consequently, further assessment of the detection systems used for ADV, HCoV and EV/HRV should be considered.

## Supplementary Material

PCR/RT-PCR protocols for virus detecting. Primer sequences and amplification programs for PCR/RT-PCR or nested PCR were provided in detail. Preparations of PCR or RT-PCR reactions and the size of corresponding PCR products were also involved.

## Figures and Tables

**Table 1:** Main clinical symptoms and blood test values of 596 patients tested.

Age (median; range)	31 (14–88)
Gender (M/F)	304/292
Body temperature (median; range)	38.61 (38–41)

Patients with clinical symptoms	Cases

Fever	521
Headache	286
Sore throat	228
Chills	150
Nasal discharge	121
Cough	113
Muscle soreness	61
Sneeze	61
Trembling	60
Arthralgia	42
Expectoration	32
Nausea	22
Diarrhea	19
Tears	17
Vomiting	11

Blood test	Value

WBCs (median; range), 10*e*9/L	8.22 (1.39–29.1)
LYM (median; range) 10*e*9/L	1.47 (0.17–24.2)
NEU (%) (median; range)	70.77 (1.89–91.5)
LYM (%) (median; range)	17.09 (0.173–80)
HGB (median; range) g/L	139.05 (13.5–184)
PLT (median; range) 10*e*9/L	221.83 (18.4–671)

WBCs: white blood cells; LYM: lymphocytes; NEU: neutrophils; HGB: haemoglobin; PLT: platelet.

**Table 2:** Spectrum of respiratory virus infections according to age groups.

	14–25 years	26–65 years	≥66 years	Total
Patients number	266	303	27	596
Total positives (number/%)	114/42.86	111/36.63	6/22.22	231/38.76
Single infections (number/%)	94/35.33	103/33.99	6/22.22	203/34.06
Multiplex infections (number/%)	20/7.52	8/2.64	0	28/4.70
IFV	62/23.31	60/19.80	2/7.41	124/20.81
IFVA	22/8.27	22/7026	2/7.41	46/7.72
IFVB	38/14.26	37/12.21	0	75/12.58
IFVC	2/0.75	1/0.33	0	3/0.50
HBoV	27/10.15	21/6.93	1/3.70	49/8.22
HRV	18/6.77	11/3.63	1/3.70	30/5.03
EV	13/4.88	9/2.97	0/0	22/3.69
RSV	7/2.63	6/1.98	2/7.41	15/2.52
PIV	1/0.38	4/1.32	0	5/0.84
HMPV	0	5/1.65	0	5/0.84
ADV	4/1.50	1/0.33	0	5/0.84
HCoV	2/0.75	2/0.66	0	4/0.67

IFV: influenza virus; HBoV: human bocavirus; HRV: human rhinovirus; EV: enterovirus; RSV: respiratory syncytial virus; PIV: parainfluenza virus; HMPV: human metapneumovirus; ADV: adenovirus; HCoV: human coronavirus.

**Table 3:** Codetection of respiratory viruses in adults with acute respiratory tract infections.

Mixed virus	14–25 years	26–65 years
IFVA + HBoV	6	0
HRV + HBoV	0	6
EV + HBoV	4	0
IFVA + HRV	3	1
EV + ADV	1	0
EV + IFVC	1	0
HMPV + RSV	1	0
IFVB + HBoV	1	0
IFVB + RSV	0	1
IFVB + HRV	1	0
IFVB + HCoV	1	0
PIV + IFVB + HBoV	1	0

IFV: influenza virus; HBoV: human bocavirus; HRV: human rhinovirus; EV: enterovirus; RSV: respiratory syncytial virus; PIV: parainfluenza virus; HMPV: human metapneumovirus; ADV: adenovirus.

**Table 4:** Respiratory virus detection rate in adult patients with ARTIs from Beijing, Shanghai, and Jinan.

Virus	Beijing [11]	Beijing [19]	Shanghai [10]	Jinan
	(2005–2007)	(2010-2011)	(2009-2010)	(2009-2010)
Number of samples	5808	416	1576	596
	34.60%	52.88%	27.47%	38.76%
IFV (A + B + C)	19.30%	16.83%	12.24%	20.81%
IFVA	16.05%	16.11%	11.61%	7.72%
IFVB	3.05%	0.72%	0.63%	12.58%
IFVC	0.26%	N.D.	N.D.	0.50%
ADV	0.90%	11.30%	1.14%	0.84%
PIV	4.30%	0.96%	1.33%	0.84%
Picornavirus/HRV + EV	9.70%	17.79%	7.23%	8.72%
RSV	0.50%	0.00%	0.63%	2.52%
HMPV	0.30%	2.16%	0.32%	0.84%
HCoV	1.10%	11.79%	0.63%	0.67%
HBoV	N.D.	N.D.	0.25%	8.22%

N.D.: not detected.

IFV: influenza virus; HBoV: human bocavirus; HRV: human rhinovirus; EV: enterovirus; RSV: respiratory syncytial virus; PIV: parainfluenza virus; HMPV: human metapneumovirus; ADV: adenovirus.
