# Supplementary material for: Viral Aetiology in Adults with Acute Upper Respiratory Tract Infection in Jinan, Northern China
Source: Clin Dev Immunol. 2013 Apr 15;2013:869521. doi: 10.1155/2013/869521 (PMC3649347; doi:10.1155/2013/869521)
Supplement: Supplementary file 1 — PCR/RT-PCR protocols for virus detecting. Primer sequences and amplification programs for PCR/RT-PCR or nested PCR were provided in detail. Preparations of PCR or RT-PCR reactions and the size of corresponding PCR products were also involved. [file 869521.f1.doc]

**Supplementary S1**

**Touch-down/nested PCR detection for human bocavirus (HBoV)**

**First round of nested PCR**

**PCR reaction**

1. Prepare the reaction mixture according to the tables below (Ex Taq, Takara, Cat.No. DRR001A～DRR001C).

| Component | Volume for 1 reaction | Volume for 50 reactions |
| --- | --- | --- |
| RNAse/DNAse free water | 15μl | 750μl |
| 10× ExBuffer (with MgCI2) | 2μl | 100μl |
| dNTP 10mmol/L | 0.4μl | 20μl |
| Primer AK-VP-F1 50μmol/L | 0.2μl | 10μl |
| Primer AK-VP-R1 50μmol/L | 0.2μl | 10μl |
| Eⅹ Taq 5U/μl | 0.2μl | 10μl |

2. Pipette 18μl of this mix into each well.

3. Pipette 2μl of extracted DNA template into each well, according to the experimental plate set up. For negative control, 2μl of RNAse/DNAse free water was added into each well. The final volume is 20μl.

**Amplification Protocol**

|  | Step | Time | Temperature |
| --- | --- | --- | --- |
|  | Denaturation | 3min | 94°C |
| 10 cycles | Denaturation | 35sec | 94°C |
|  | Annealing | 1min | 58°C(decreased by 0.5°C every cycle) |
|  | Extension | 1min | 72°C |
| 30cycles | Denaturation | 30sec | 94°C |
|  | Annealing | 45sec | 54°C |
|  | Extension | 45sec | 72°C |
|  | Extension | 10min | 72°C |

**Second round of nested PCR**

PCR reaction system was the same as the first round of nested PCR, the reaction mixture for the second round was added with 2µl PCR product from the first round. PCR primer paris of AK-VP-F2 and AK-VP-R2 were added instead of AK-VP-F1 and AK-VP-R1. PCR amplification protocol was also the same as the first round. PCR products were visualized following electrophoresis on 2% agarose gel.

Nested PCR primers for human Bocavirus

| Primers | Sequences | Gene | Size |
| --- | --- | --- | --- |
| AK-VP-F1 | 5’-CGCCGTGGCTCCTGCTCT-3’ | VP1/VP2 | 611bp |
| AK-VP-R1 | 5’-TGTTCGCCATCACAAAAGATGTG-3’ |  |  |
| AK-VP-F2 | 5’-GGCTCCTGCTCTAGGAAATAAAGAG-3’ |  | 576bp |
| AK-VP-R2 | 5’CCTGCTGTTAGGTCGTTGTTGTATGT-3’ |  |  |

**PCR detection for human adenovirus (ADV)**

**PCR reaction**

1. Prepare the reaction mixture according to the tables below (Ex Taq, Takara, Cat.No. DRR001A～DRR001C).

| Component | Volume for 1 reaction | Volume for 50 reactions |
| --- | --- | --- |
| RNAse/DNAse free water | 14.5μl | 725μl |
| 10×Ex Buffer (with MgCI2) | 2μl | 100μl |
| dNTP 10mmol/L | 0.4μl | 20μl |
| Primer 1-ADVF 50μmol/L | 0.4μl | 20μl |
| Primer 1-ADVR 50μmol/L | 0.4μl | 20μl |
| Eⅹ Taq 5U/μl | 0.3μl | 15μl |

2. Pipette 18μl of this mix into each well.

3. Pipette 2μl of extracted DNA template into each well, according to the experimental plate set up. For negative control, 2μl of RNAse/DNAse free water was added into each well. The final volume is 20μl.

4. PCR products were visualized following electrophoresis on 2% agarose gel.

**Amplification Protocol**

|  | Step | Time | Temperature |
| --- | --- | --- | --- |
|  | Denaturation | 3min | 94°C |
| 35 cycles | Denaturation | 30sec | 94°C |
|  | Annealing | 30sec | 55°C |
|  | Extension | 45sec | 72°C |
|  | Extension | 10min | 72°C |

**RT-PCR primers for human adenovirus**

| Primers | Sequences | Gene | Size |
| --- | --- | --- | --- |
| 1-ADVF | 5’-GCCSCARTGGKCWTACATGCACATC-3’ | hexon | 301bp |
| 1-ADVR | 5’-CAGCACSCCICGRATGTCAAA-3’ |  |  |

**RT-PCR detection for human coronavirus (HCoV)**

**RT-PCR reaction**

1. Prepare the reaction mixture according to the tables below (Invitrogen One-step RT-PCR kit, Cat.No.12574-026)

| Component | Volume for 1 reaction | Volume for 50 reactions |
| --- | --- | --- |
| RNAse/DNAse free water | 6.4μl | 320μl |
| 2×Reaction Mix | 10μl | 500μl |
| Primer HcoVFc 50μmol/L | 0.4μl | 20μl |
| Primer HcoVRc 50μmol/L | 0.4μl | 20μl |
| SuperScriptTM Ⅲ One-step RT/platiinumR Taq mix | 0.8μl | 40μl |

2. Pipette 18μl of this mix into each well.

3. Pipette 2μl of extracted DNA template into each well, according to the experimental plate set up. For negative control, 2μl of RNAse/DNAse free water was added into each well. The final volume is 20μl.

4. RT-PCR products were visualized following electrophoresis on 2% agarose gel.

**Amplification Protocol**

|  | Step | Time | Temperature |
| --- | --- | --- | --- |
|  | Reverse transcription | 45min | 48°C |
|  | Denaturation | 3min | 94°C |
| 40 cycles | Denaturation | 30sec | 94°C |
|  | Annealing | 30sec | 55°C |
|  | Extension | 45sec | 72°C |
|  | Extension | 10min | 72°C |

**RT-PCR primers for human coronavirus**

| Primers | Sequences | Gene | Size |
| --- | --- | --- | --- |
| HcoVFc | 5’-GGTTGGGACTATCCTAAGTGTGA-3’ | Pol | 440bp |
| HcoVRc | 5’-CCATCATCAGATAGAATCATCATA-3’ |  |  |

**RT-PCR detection for human metapneumovirus (MPV)**

**RT-PCR reaction**

1. Prepare the reaction mixture according to the tables below (Invitrogen One-step RT-PCR kit, Cat.No.12574-026)

| Component | Volume for 1 reaction | Volume for 50 reactions |
| --- | --- | --- |
| RNAse/DNAse free water | 6.4μl | 320μl |
| 2×Reaction Mix | 10μl | 500μl |
| Primer HcoVFc 50μmol/L | 0.4μl | 20μl |
| Primer HcoVRc 50 μmol/L | 0.4μl | 20μl |
| SuperScriptTM Ⅲ One-step RT/platiinumR Taq mix | 0.8μl | 40μl |

2. Pipette 18μl of this mix into each well.

3. Pipette 2μl of extracted DNA template into each well, according to the experimental plate set up. For negative control, 2μl of RNAse/DNAse free water was added into each well. The final volume is 20μl.

4. RT-PCR products were visualized following electrophoresis on 2% agarose gel.

**Amplification Protocol**

|  | Step | Time | Temperature |
| --- | --- | --- | --- |
|  | Reverse transcription | 45min | 48°C |
|  | Denaturation | 3min | 94°C |
| 40 cycles | Denaturation | 30sec | 94°C |
|  | Annealing | 30sec | 54°C |
|  | Extension | 1min | 72°C |
|  | Extension | 10min | 72°C |

**RT-PCR primers for human metapneumovirus**

| Primers | Sequences | Gene | Size |
| --- | --- | --- | --- |
| MPV P F | 5’-TyAACATTGCwACAGCAGGACC-3’ | P | 247bp |
| MPV P R | 5’-CTTCWGATTCWCCRCTTGTGCT-3’ |  |  |

**Multiplex RT-PCR detection for human parainfluenza (PIV), rhinovirus (RoV) and enterovirus (EnV)**

**First round of RT-PCR**

**RT-PCR reaction**

1. Prepare the reaction mixture according to the tables below (Invitrogen One-step RT-PCR kit, Cat.No.12574-026).

| Component | Volume for 1 reaction | Volume for 50 reactions |
| --- | --- | --- |
| RNAse/DNAse free water | 6.24μl | 312μl |
| 2ⅹReaction Mix | 10μl | 500μl |
| SuperScriptTM Ⅲ One-step RT/platiinumR Taq mix | 0.8μl | 40μl |
| Primer 1PIV13 50μmol/L | 0.16μl | 8μl |
| Primer 2PIV13 50μmol/L | 0.16μl | 8μl |
| Primer 1PIV2 50μmol/L | 0.16μl | 8μl |
| Primer 1PIV4 50μmol/L | 0.16μl | 8μl |
| Primer 2PIV24 50μmol/L | 0.16μl | 8μl |
| Primer 1-EV/RV 50μmol/L | 0.08μl | 4μl |
| Primer 2-EV/RV 50μmol/L | 0.08μl | 4μl |

2. Pipette 18μl of this mix into each well.

3. Pipette 2μl of extracted DNA template into each well, according to the experimental plate set up. For negative control, 2μl of RNAse/DNAse free water was added into each well. The final volume is 20μl.

4. RT-PCR products were visualized following electrophoresis on 2% agarose gel.

**Amplification Protocol**

|  | Step | Time | Temperature |
| --- | --- | --- | --- |
|  | Reverse transcription | 45min | 48°C |
|  | Denaturation | 3min | 94°C |
| 35 cycles | Denaturation | 30sec | 94°C |
|  | Annealing | 1min | 55°C |
|  | Extension | 30sec | 72°C |
|  | Extension | 10min | 72°C |

**Second round of nested PCR**

Nested PCR reaction

1. Prepare the reaction mixture according to the tables below (Ex Taq, Ex Taq, Takara, Cat.No. DRR001A～DRR001C)

| Component | Volume for 1 reaction | Volume for 50 reactions |
| --- | --- | --- |
| RNAse/DNAse free water | 14.38μl | 719μl |
| 10 x Ex Buffer(with MgCI2) | 2μl | 100μl |
| dNTP 10 mmol/L | 0.5μl | 25μl |
| Primer 3PIV13 50μmol/L | 0.16μl | 8μl |
| Primer 4PIV1 50μmol/L | 0.16μl | 8μl |
| Primer 4PIV3 50μmol/L | 0.16μl | 8μl |
| Primer 3PIV24 50μmol/L | 0.16μl | 8μl |
| Primer 4PIV2 50μmol/L | 0.16μl | 8μl |
| Primer 3-EV/RV 50μmol/L | 0.08μl | 4μl |
| Primer 4-EV/RV 50μmol/L | 0.08μl | 4μl |
| Ex Taq 5U/ul | 0.1μl | 5μl |

2. Pipette 18μl of this mix into each well.

3. Pipette 2μl of RT-PCR product from the first round into each well, according to the experimental plate set up. For negative control, 2μl of RNAse/DNAse free water was added into each well. The final volume is 20μl.

4. Multiplex PCR products were visualized following electrophoresis on 2% agarose gel.

**Amplification Protocol**

|  | Step | Time | Temperature |
| --- | --- | --- | --- |
|  | Denaturation | 4min | 94°C |
| 35 cycles | Denaturation | 30sec | 94°C |
|  | Annealing | 1min | 55°C |
|  | Extension | 30sec | 72°C |
|  | Extension | 10min | 72°C |

Multiplex PCR primers for parainfluenza,rhinovirus and enterovirus

| Primers | Sequences | Size |
| --- | --- | --- |
| 1PIV13 | 5’-AGGWTGYSMRGATATAGGRAARTCAT-3’ | PIV1:439bp  PIV2:297bp  PIV3:390bp  PIV4:174bp |
| 2PIV13 | 5’-CTWGTATATATATRTAGATCTTKTTRCCTAGT-3’ |
| 1PIV2 | 5’-TAATTCCTCTTAAAATTGACAGTATCGA-3’ |
| 1PIV4 | 5’-ATCCAGARRGACGTCACATCAACTCAT-3’ |
| 2PIV24 | 5’-TRAGRCCMCCATAYAMRGGAAATA-3’ |
| 3PIV13 | 5’-ACGACAAYAGGAARTCATGYTCT-3’ |
| 4PIV1 | 5’-GACAACAATCTTTGGCCTATCAGATA-3’ |
| 4PIV3 | 5’-GAGTTGACCATCCTYCTRTCTGAAAAC-3’ |
| 3PIV24 | 5’-CYMAYGGRTGYAYTMGAATWCCATWCCATCATT-3’ |
| 4PIV2 | 5’-GCTAGATCAGTTGTGGCATAATCT-3’ |
| 4PIV4 | 5’-TGACTATRCTCGACYTTRAAATAAGG-3’ |
| 1-EV/RV | 5’-CTCCGGCCCCTGAATRYGGCTAA-3’ | EV:226bp  (200bp-232bp)  RV:110bp  (100bp-120bp) |
| 2-EV/RV | 5’-TCIGGIARYTTCCASYACCAICC-3’ |
| 3-EV/RV | 5’-ACCRASTACTTTGGGTRWCCGTG-3’ |
| 4-EV/RV | 5’-CTGTGTTGAWACYTGAGCICCCA-3’ |

**Multiplex RT-PCR detection for human influenza (Flu) and respiratory syncytial virus (RSV)**

**First round of RT-PCR**

**RT-PCR reaction**

1. Prepare the reaction mixture according to the tables below (Invitrogen One-step RT-PCR kit, Cat.No.12574-026).

| Component | Volume for 1 reaction | Volume for 50 reactions |
| --- | --- | --- |
| RNAse/DNAse free water | 6.2μl | 310μl |
| 2ⅹReaction Mix | 10μl | 500μl |
| SuperScriptTM Ⅲ One-step RT/platiinumR Taq mix | 0.8μl | 40μl |
| Primer FluAC1 50μmol/L | 0.2μl | 10μl |
| Primer FluB1 50μmol/L | 0.2μl | 10μl |
| Primer FluABC2 50μmol/L | 0.2μl | 10μl |
| Primer RSVAB1 50μmol/L | 0.2μl | 10μl |
| Primer RSVAB2 50μmol/L | 0.2μl | 10μl |

2. Pipette 18μl of this mix into each well.

3. Pipette 2μl of extracted DNA template into each well, according to the experimental plate set up. For negative control, 2μl of RNAse/DNAse free water was added into each well. The final volume is 20μl.

4. RT-PCR products were visualized following electrophoresis on 2% agarose gel.

**Amplification Protocol**

|  | Step | Time | Temperature |
| --- | --- | --- | --- |
|  | Reverse transcription | 45min | 48°C |
|  | Denaturation | 3min | 94°C |
| 35 cycles | Denaturation | 30sec | 94°C |
|  | Annealing | 1min | 55°C |
|  | Extension | 30sec | 72°C |
|  | Extension | 10min | 72°C |

**Second round of nested PCR**

**Nested PCR reaction**

1. Prepare the reaction mixture according to the tables below (Ex Taq, Takara, Cat.No. DRR001A～DRR001C).

| Component | Volume for 1 reaction | Volume for 50 reactions |
| --- | --- | --- |
| RNAse/DNAse free water | 13.8μl | 690μl |
| 10 x Ex Buffer(with MgCI2) | 2μl | 100μl |
| dNTP 10mol/L | 0.5μl | 25μl |
| Primer FluAB3 50μmol/L | 0.2μl | 10μl |
| Primer FluC3 50μmol/L | 0.2μl | 10μl |
| Primer FluAC3 50μmol/L | 0.2μl | 10μl |
| Primer FluB4 50μmol/L | 0.2μl | 10μl |
| Primer RSVA3 50μmol/L | 0.2μl | 10μl |
| Primer RSVA4 50μmol/L | 0.2μl | 10μl |
| Primer RSVB3 50μmol/L | 0.2μl | 10μl |
| Primer RSVB4 50μmol/L | 0.2μl | 10μl |
| Ex Taq 5U/μl | 0.1μl | 5μl |

2. Pipette 18μl of this mix into each well.

3. Pipette 2μl of extracted DNA template into each well, according to the experimental plate set up. For negative control, 2μl of RNAse/DNAse free water was added into each well. The final volume is 20μl.

4. Multiplex PCR products were visualized following electrophoresis on 2% agarose gel.

**Amplification Protocol**

|  | Step | Time | Temperature |
| --- | --- | --- | --- |
|  | Denaturation | 4min | 94°C |
| 35 cycles | Denaturation | 30sec | 94°C |
|  | Annealing | 1min | 55°C |
|  | Extension | 30sec | 72°C |
|  | Extension | 10min | 72°C |

**Multiplex PCR primers for human innfluenza and respiratory syncytial virus**

| Primers | Sequences | Size |
| --- | --- | --- |
| RT-PCR | ’ |  |
| FluAC1 | 5’-GAACTCRTYCYWWATSWCAAWGRRGAAAT-3’ |  |
| FluB1 | 5’-ACAGAGATAAAGAAGAGCGTCTACAA-3’ |
| FluABC2 | 5’-ATKGCGCWYRAYAMWCTYARRTCTTCAWAIGC-3’ |
| RSVAB1 | 5’ATGGAGYTGCYRATCCWCARRRCAARTGCAAT-3’ |
| RSVAB2 | 5’-AGGTGTWGTTACCCTGCATTRACACTRAATTC-3’ |
| Nested-PCR |  |  |
| FluAB3 | 5’-GATCAAGTGAKMGRRAGYMGRAAYCCAGG-3’ | Influenza  (A)301bp  (B)226bp  (C)111bp |
| FluC3 | 5’-AAATTGGAATTTGTTCCTTTCAAGGGACA-3’ |
| FluAC3 | 5’-TCTTCAWATGCARSWSMAWKGCATGCCATC-3’ |
| FluB4 | 5’-CTTAATATGGAAACAGGTGTTGCCATATT-3’ |
| RSVA3 | 5’-TTATACACTCAACAATRCCAAAAAWACC-3’ | RSV  (A)363bp  (B)611bp |
| RSVA4 | 5’-AAATTCCCTGGTAATCTCTAGTAGTAGTAGTCTGT-3’ |
| RSVB3 | 5’-ATCTTCCTAACTCTTGCTRTTAATGCATTG-3’ |
| RSVB4 | 5’-GATGCGACAGCTCTGTTGATTACTATG-3’ |
